# Intraoperative Atrial Fibrillation in Non-Cardiac Surgery: A Narrative Review of Risk Factors and Management Strategies

**DOI:** 10.31083/RCM51310

**Published:** 2026-07-21

**Authors:** Amin Hasheminia, Kavi Gupta, Margo Kaminska, Maria Luz Garagiola, Amin Meghdadi, Norberto Bornancini, Alejandro L. Botbol, José Elizardo Llorente Rodríguez, Zier Zhou, Fausto Heredia Villacreses, Adrian Baranchuk

**Affiliations:** ^1^Faculty of Medicine and Health Sciences, McGill University, Montreal, QC H3G 2M1, Canada; ^2^Faculty of Health Sciences, School of Medicine, Queen’s University, Kingston, ON K7L 3N6, Canada; ^3^Department of Biomedical and Molecular Sciences, Queen’s University, Kingston, ON K7L 3N6, Canada; ^4^Division of Cardiology, Kingston Health Sciences Centre, Queen’s University, Kingston, ON K7L 2V7, Canada; ^5^Faculty of Medicine, Universidad Abierta Interamericana (UAI), C1147AAU Buenos Aires, Argentina; ^6^Universidad De Especialidades Espíritu Santo, 092301 Guayaquil, Ecuador; ^7^Faculty of Medicine, Queen’s University, Kingston, ON K7L 3N6, Canada; ^8^Servicio de Medicina Intensiva, Servicio de Cardiología, Hospital Clínica San Francisco, 090150 Guayaquil, Ecuador

**Keywords:** atrial fibrillation, intraoperative period, monitoring, intraoperative, electrocardiography, perioperative care, surgical procedures, operative, anticoagulants, electric countershock

## Abstract

Atrial fibrillation (AF) is the most prevalent cardiac arrhythmia. A less studied presentation of AF is intraoperative atrial fibrillation (IOAF), which is defined as AF occurring during surgery. IOAF has important clinical implications, as this condition is associated with increased morbidity and mortality, even in non-cardiac surgical procedures. A narrative review of studies published between 1990 and 2025 was conducted using PubMed, ScienceDirect, and the Cochrane Library to evaluate the risk factors and management of IOAF in non-cardiac surgery. Reported IOAF prevalence varies by procedure type, ranging from 1% to 5% in most non-cardiac surgeries. Across non-cardiac surgical procedures, three IOAF risk domains emerge: patient profile, including age, prior AF, and structural heart disease; the invasiveness of the procedure; acute triggers, including hypotension and electrolyte imbalance. Management of IOAF begins with rapid assessment of hemodynamic stability and identification of surgical triggers. In hemodynamically unstable patients, particularly those with myocardial ischemia or pulmonary edema, immediate synchronized direct-current cardioversion (DCCV) is indicated. Conversely, in hemodynamically stable patients, the “3As” management framework is usually followed: first, acute triggers are addressed; then, the AF rate or rhythm is controlled; finally, anticoagulation is considered using the CHA_2_DS_2_-VASc score. Across the spectrum of surgical procedures, IOAF is best documented in cardiac surgery, where guidelines for the associated management and prevention are readily available. To further investigate IOAF in non-cardiac surgery, adopting standardized definitions is critical to advancing research and improving management.

## 1. Introduction

Atrial fibrillation (AF) is the most prevalent sustained cardiac arrhythmia, serving as a notable marker of cardiovascular and electrophysiological instability [1]. A significant presentation of AF is intraoperative AF (IOAF), which encompasses AF occurring during surgery [2,3]. Although a more uncommon presentation of AF, this complication can lead to hemodynamic instability, myocardial ischemia, oxygenation concerns, pulmonary complications, and increased perioperative morbidity and mortality [2,4]. Notably, high-risk operations are most prone to IOAF, specifically non-cardiac surgeries with prolonged anesthesia [2,4]. IOAF can arise in thoracic procedures such as lung resection (10–20% of lobectomies and 30–40% of pneumonectomies), esophagectomies (12–37%), and vascular surgeries (3.7%–4.7%) [5,6,7,8,9,10,11]. While modern cardiac surgical literature, informed by decades of patient data, has produced substantial treatment strategies and protocols to alleviate intraoperative AF during cardiac surgery, there is limited guidance on its management, particularly during non-cardiac surgery [12,13]. Since evidence outside cardiovascular surgery is largely observational, focused guidelines surrounding AF for non-cardiac surgery are mainly extrapolated from perioperative, postoperative, or chronic AF cohorts, with most of these guidelines applying to broad cardiac management as opposed to procedure-specific management of IOAF [14,15]. This gap leaves clinicians without standardized frameworks for identifying patients at risk, implementing preventive strategies, and guiding intraoperative management. This narrative review synthesizes current evidence on IOAF across a broad range of non-cardiac surgical procedures, aiming to clarify risk factors, clinical implications, and practical management considerations while positioning IOAF as a manifestation of intraoperative physiological stress.

## 2. Search Strategy and Study Selection

A comprehensive electronic literature search was conducted across PubMed, Cochrane Library, and ScienceDirect databases. Keywords included a combination of atrial fibrillation, perioperative, postoperative, intraoperative, non-cardiac surgery, prevention, monitoring, and epidemiology. Standard Boolean operators and Medical Subject Headings (MeSH) terms were applied to enhance sensitivity and specificity in our search. Hand searches for the reference lists of the included studies were also conducted to identify further pertinent publications. These included studies that met the following criteria: publication dates ranging from January 1990 through November 2025, a focus on AF during or after non-cardiac surgery and the use of quantitative (e.g., case report, case-control, cross-sectional, cohort or randomized clinical trial) or qualitative study designs. The exclusion criteria included studies focusing solely on cardiac surgery. Definitions of intraoperative atrial fibrillation (IOAF) varied, and it was generally identified by intraoperative electrocardiographic monitoring, with some studies using continuous electrocardiography (ECG) and others using confirmatory 12-lead ECG. Minimum episode duration also differed, most commonly ranging from 30 seconds to 1 minute. Multiple reviewers (AH, KG, MG) independently screened article titles and abstracts to assess relevance. A total of 1467 articles were initially identified. Full texts of the shortlisted studies were retrieved and reviewed in detail. After applying the inclusion and exclusion criteria, 41 articles were ultimately included in the final manuscript. Any discrepancies in article selection were resolved through a consensus discussion with a senior reviewer (AB).

## 3. General Principles of Intraoperative AF Management

**Fig. 1.**
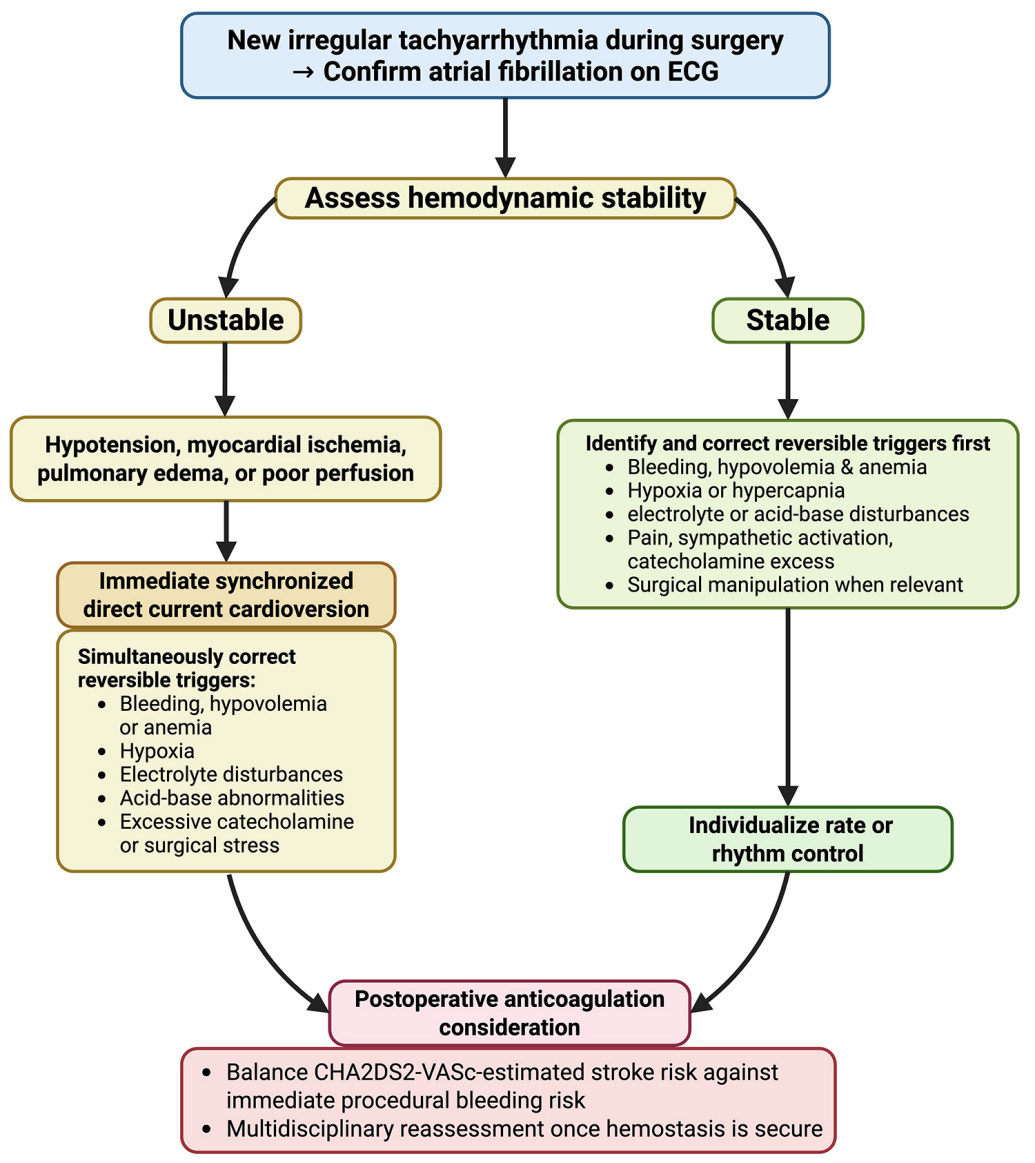
**Proposed perioperative management algorithm for intraoperative atrial fibrillation (IOAF) during non-cardiac surgery**. ECG, electrocardiography.

As illustrated by Fig. 1, general management of IOAF begins with rapid assessment of hemodynamic stability and identification of any reversible surgical triggers [16,17]. Hemodynamic instability is shown by hypotension, ongoing myocardial ischemia, and pulmonary edema, and it requires immediate synchronized direct current cardioversion (DCCV) (Class I, Level B) [1,16,17]. In contrast, hemodynamically stable patients usually follow the “3As” management system, which first addresses the acute triggers, then controls atrial fibrillation rate or rhythm, and then considers anticoagulation [16].

During surgery, the initial priority in patients who develop AF is to exclude active bleeding. Intraoperative tachycardia is frequently a compensatory response because of hypovolemia, anemia, or hemodynamic compromise [16,17]. Acute blood loss promotes catecholamine release and disrupts autonomic balance. This stress response increases the risk of atrial fibrillation, so treating AF without addressing the bleeding may suppress a critical compensatory mechanism [17]. In addition, a systematic evaluation for reversible precipitants should follow. Electrolyte imbalance (mainly affecting potassium and magnesium), hypoxia, acid-base disturbances, or heightened sympathetic hyperactivity due to inadequate analgesia are common intraoperative triggers and should be promptly corrected [16,17,18]. Then, if the patient’s blood pressure (BP) and circulation are stable, physicians should first focus on slowing the heart rate (HR) to 110 beats per minute [16]. If the patient has a preserved left ventricular ejection fraction (LVEF >40%), then intravenous beta-blockers (e.g., metoprolol or esmolol) or non-dihydropyridine calcium channel blockers (e.g., diltiazem or verapamil) can be used to slow the HR [16,19].

However, in patients with reduced left ventricular systolic function, non-dihydropyridine calcium channel blockers (diltiazem or verapamil) should be avoided, as their negative inotropic effect may precipitate acute cardiac decompensation (Class III: Harm) [19]. In that case, it is safer to use amiodarone (Class IIb, Level B-NR), which can help control HR without significantly weakening the heart. Finally, drugs such as Digoxin usually do not work well during surgery because stress hormones such as adrenaline counteract Digoxin’s effects [16,19].

If the cardiac rhythm needs to be restored quickly, such as in situations involving heart failure or severe ischemia, physicians may use a different approach [16]. If the patient does not have left ventricular dysfunction or structural heart disease, intravenous flecainide or propafenone can quickly restore the heart rhythm in more than half of cases [16,19]. Another option is to use procainamide, but it is not as effective and causes hypotension in 5–12% of patients [1,19]. The decision to initiate anticoagulation should involve all specialties’ physicians involved in the procedure and the patient to accurately weigh the patient’s substrate-based stroke risk (using CHA_2_DS_2_-VASc scores) against the immediate surgical bleeding risk [17].

### 3.1 Intraoperative AF in Thoracic Surgery

Studies reporting on IOAF for these procedures are summarized in Table 1 (Ref. [10,20,21,22,23,24,25]). Across large lung‑resection cohorts, IOAF occurs in about 1–3% of cases. In 10,563 lung operations (mostly open), Wu et al. (2012) [20] found IOAF in 3.27%; open surgery had 3.81% vs 1.57% with video-assisted thoracoscopic surgery (VATS) (adjusted odds ratio [OR] 2.07). In 14,986 thoracoscopic anatomic resections, Tong et al. (2021) [21] reported IOAF in 1.2% (177/14,986). A prospective open thoracotomy series including lung and esophageal cancer found IOAF in 12.5% (18/144) with continuous ECG, reflecting a high‑risk population [22].

**Table 1.** **Thoracic surgery studies: design/sample, procedures, intraoperative atrial fibrillation (IOAF) definition, incidence, and associated risk factors**.

Study	Design/n	Procedure(s)	IOAF definition	IOAF incidence	Independent risk factors/associations
Wu et al., 2012 [20]	Retrospective, n = 10,563	Lung resections (open & VATS)	AF during surgery on continuous ECG	3.27%	Age, male sex, lung cancer, GA+paravertebral block, open surgery, ≥lobectomy, longer OR time; 40.7% during lymph node dissection
Xie et al., 2018 [22]	Prospective, n = 144	Lung & esophageal thoracotomy	AF ≥30 s intraop ECG	12.5%	Alcohol use, prior chemotherapy, higher HR during one‑lung ventilation; lower BMI, smaller tidal volumes
Tong et al., 2021 [21]	Retrospective, n = 14,986	Thoracoscopic anatomic lung surgery	AF during surgery	1.2%	Age ≥60, male sex, diabetes, tumor ≥1.4 cm, nodal involvement, lobectomy, right‑sided surgery; IOAF → longer ICU & LOS
Jiang et al., 2023 [10]	Case‑control, n = 80	Lung surgery	AF during surgery	50% in high‑risk model	Left‑atrial enlargement, QRS prolongation, higher ASA, open surgery—strong predictors
Hahm et al., 2007 [23]	Retrospective, n = 427	Transthoracic esophagectomy	Any non‑sinus intraop rhythm	17.1% arrhythmias	Cardiac disease, poor PFTs, cervical anastomosis, high CVP, higher ephedrine dose; 37% recurrence in 3 days; associated with pulmonary & anastomotic complications
Malhotra et al., 2006 [24]; Nikbakhsh et al., 2012 [25]	Prospective, small cohorts	Transhiatal esophagectomy	Holter‑detected arrhythmias during mediastinal dissection	AF infrequent; total arrhythmias ~60–80%	Mediastinal manipulation → hypotension, brady/tachyarrhythmias; mostly self‑limited

Abbreviations: AF, atrial fibrillation; IOAF, intraoperative atrial fibrillation; n, sample size; VATS, video-assisted thoracoscopic surgery; ECG, electrocardiogram; GA, general anesthesia; OR, operating room; HR, heart rate; BMI, body mass index; ICU, intensive care unit; LOS, length of stay; ASA, American Society of Anesthesiologists physical status classification; PFTs, pulmonary function tests; CVP, central venous pressure.

Shared predictors across ≥2 studies include age ≥60 years and male sex (ORs ≈ 1.9 and 2.3–3.0), more extensive resection (lobectomy/pneumonectomy vs wedge/segment, OR ≈ 3–6), and open approach compared to thoracoscopic approach [20,21]. Xie et al. (2018) [22] additionally identified lung/esophageal malignancy, pre‑operative chemotherapy (OR 4.0), heavy alcohol use (OR 5.3) and higher HR during one‑lung ventilation (OR 1.09 per bpm) as independent predictors. Jiang et al. (2023) [10] showed that left‑atrial enlargement, QRS ≥112.5 ms, higher American Society of Anesthesiologists physical status classification (ASA) class and open surgery formed a four‑variable model in which all patients with ≥2 features developed IOAF. Timing analyses further support a mechanistic link between surgical manipulation and IOAF. Wu et al. (2012) [20] reported that 40.7% of IOAF episodes occurred during mediastinal/hilar lymph‑node dissection, 23.1% during parenchymal resection, and 28.0% during hemostasis, implicating mediastinal manipulation and autonomic perturbation as key triggers. Additionally, certain cohorts linked intraoperative arrhythmias to cardiopulmonary reserve and intraoperative hemodynamics. Hahm et al. (2007) [23] reported intraoperative arrhythmias in 17.1% of 427 transthoracic esophagectomies and identified heart disease, poor pulmonary function tests (PFTs), cervical anastomosis, elevated central venous pressure (CVP), and higher ephedrine dose as independent predictors, with 37.2% recurring within 3 postoperative days. Malhotra et al. (2006) [24] and Nikbakhsh et al. (2012) [25] found that arrhythmias rise sharply during mediastinal manipulation in transhiatal esophagectomy and are typically transient (often ectopy/bradycardia).

In terms of clinical impact, Tong et al. (2021) [21] found that IOAF was associated with longer intensive care unit (ICU) stay (median 28 vs 24 h) and hospital length of stay (LOS) (6 vs 5 days), although no clear excess of major complications was observed after propensity-matching. Preventive strategies remain inconsistent. In 416 patients with transient IOAF during lung/esophageal surgery, intraoperative amiodarone shortened postoperative AF duration (median 1.1 vs 1.8 days) but increased hypotension (6.6% severe) without improving on-table cardioversion (~23% in both groups) [20]. Aoyama et al. (2016) [26] found that low‑dose landiolol during lung resection did not reduce postoperative atrial fibrillation (POAF) (20% vs 16%), and no IOAF occurred in either arm. Holistically, it is important to observe these factors to mitigate the emergence of IOAF in lung and esophageal surgeries, distinguishing it as a procedure-dependent electrophysiologic response rather than a random complication.

### 3.2 Intraoperative AF in General Surgery and Transplantation

Studies reporting on IOAF during general surgery are summarized in Table 2 (Ref. [12,27,28,29,30,31,32]). Prospective data in endocrine and transplant surgery suggest that IOAF is uncommon but clinically meaningful. In 1252 euthyroid patients undergoing elective thyroidectomy, new‑onset IOAF occurred in 9 patients (0.72%) [33]. Pre‑operative rhythm disturbances and angina pectoris were independent predictors, and those who developed IOAF had a markedly higher rate of postoperative AF (33% vs 1.5%), indicating that even brief IOAF episodes can contribute to downstream arrhythmogenic activity [33]. In adult liver transplantation, Moon et al. (2018) [27] reported IOAF in 13 of 1059 recipients (1.2%), with most episodes occurring around graft reperfusion. Fulminant hepatic failure (odds ratio [OR] 6.84, 95% CI 1.94–24.10) and higher Model for End-Stage Liver Disease (MELD) score (OR 1.08 per point, 95% CI 1.02–1.14) independently predicted IOAF. Despite being short‑lived (often <1 week), AF was independently associated with higher post-liver-transplant mortality and graft failure [27]. A separate case report described IOAF developed within minutes of an iced‑saline bolus for transpulmonary thermodilution during liver transplantation, with AF persisting for several days but ultimately resolving with amiodarone, highlighting right‑atrial cooling as a potential reversible intraoperative trigger [28].

**Table 2.** **General surgery studies: study design, procedure, intraoperative atrial fibrillation (IOAF) definition context, and key points**.

Study/report	Design/n	Procedure	IOAF context	Key points
Jayakar et al., 2025 [12]	Case	Open nephrectomy	Unstable AF late in case	DC cardioversion with subsequent ICU care; illustrates complexity in prosthetic‑valve patient
Moon et al., 2018 [27]	Retrospective, n = 1059	Adult liver transplantation	New AF during surgery in patients with pre‑op sinus rhythm	IOAF 1.2%; risk factors: higher MELD, fulminant hepatic failure; 8/13 episodes at reperfusion; brief but IOAF independently predicted mortality (HR ≈ 5)
Li et al., 2022 [28]	Single case	Orthotopic liver transplantation	AF minutes after iced saline for thermodilution	AF with hypotension; managed with vasopressors, amiodarone; AF persisted 5 days then self‑terminated; highlights thermodilution as trigger
Bindal et al., 2019 [29]	Case	Whipple operation	IOAF during retractor use on diaphragm	Attributed to mechanical irritation of heart; resolved after removal and pharmacologic therapy
Das et al., 2017 [30]; Kumar et al., 2013 [31]	2 cases	TURP	AF during surgery under spinal anesthesia	One managed medically (esmolol, amiodarone); one required cardioversion; both underscore need for vigilance in high‑risk cardiomyopathy/renal amyloidosis
Nadir et al., 2023 [32]	Case series, n = 5	Various non‑cardiac surgeries	AF during surgery	Multiple triggers (stress, drugs, electrolytes); all patients survived; emphasizes mechanistic heterogeneity

Abbreviations: AF, atrial fibrillation; IOAF, intraoperative atrial fibrillation; n, sample size; MELD, Model for End-Stage Liver Disease; HR, hazard ratio; TURP, transurethral resection of the prostate; DC, direct current; ICU, intensive care unit.

Major abdominal cancer surgery showed a similar pattern of acute mechanical and hemodynamic provocations on a vulnerable atrium. During a pancreaticoduodenectomy (Whipple operation), Bindal et al. (2019) [29] described IOAF with hypotension coinciding with a deeply placed abdominal retractor; removal of the retractor normalized BP and HR, and sinus rhythm returned spontaneously without cardioversion. Additional case-based reports underscore that IOAF can present abruptly with hypotension across non-cardiac procedures: Das et al. [30] described sudden-onset AF during emergency clot evacuation after transurethral resection of the prostate (TURP) under spinal anesthesia, managed with pharmacologic rate/rhythm control (e.g., esmolol then amiodarone) without electrical cardioversion. A TURP case in renal amyloidosis with occult restrictive cardiomyopathy similarly developed AF with hypotension but required immediate synchronized cardioversion [31]. Winkel et al. [34] synthesized five intraoperative AF cases and emphasized perioperative stress, anesthetic exposure, electrolyte imbalance, and underlying cardiac disease as recurring contributors.

### 3.3 Intraoperative AF in Vascular Surgery

The clinically significant burden of IOAF in vascular surgery has become increasingly evident in recent literature. Studies reporting on IOAF detected during vascular surgery are summarized in Table 3 (Ref. [11,34,35]). A systematic review by Malavasi et al. [11] encompassing 44 studies and over 400,000 patients, demonstrated that approximately 11.5% of patients (95% CI 9.9–13.3) had preoperative AF. These patients experienced substantially worse outcomes, such as increased risks of stroke (OR 3.29, 95% CI 2.66–4.06) or hospital death (OR 1.61, 95% CI 1.39–1.86), displaying the role that AF plays in prognoses of this population [11]. However, new AF that begins during or after surgery is less common, and more specifically, it usually occurs during the days following surgery (post-operative AF) rather than during the operation. Overall, about 3.6% (95% CI 2–6.4%) of patients developed AF after surgery, with higher rates after open surgery (8.2%) compared to less invasive endovascular procedures (2.4%) (OR for endovascular vs open 0.35, 95% CI 0.13–0.91). Advanced age continued to be the strongest risk factor for developing AF after surgery [11].

**Table 3.** **Vascular surgery studies: design/sample, procedures, atrial fibrillation (AF) monitoring window/method, incidence, and key findings**.

Study	Design/n	Procedures	AF window & method	Incidence	Key findings
Winkel et al., 2009 [34]	Prospective, n = 317	Major vascular (AAA repair, peripheral bypass)	Continuous 12‑lead ECG from 1 day pre‑op to 2 days post	New‑onset AF 4.7%	Most episodes transient; IO/peri‑op AF independently predicted 30‑day and late CV events (HR 6.0 and 4.2)
Sposato et al., 2022 [35]	Retrospective, n = 186	Carotid endarterectomy	Continuous intra‑ & in‑hospital ECG	New AF 3.8%	Intraop hypotension only independent predictor (OR 9.6); IOAF associated with peri‑op stroke and stroke/MI composite
Malavasi et al., 2023 [11]	Systematic review & meta‑analysis (44 studies)	Vascular surgery (arterial)	History of AF/POAF	Prev. AF 11.5%; POAF 3.6%	Prior AF → higher in‑hospital death and stroke; POAF less frequent with endovascular vs open procedures

Abbreviations: AF, atrial fibrillation; AAA, abdominal aortic aneurysm; n, sample size; ECG, electrocardiogram; IOAF, intraoperative atrial fibrillation; POAF, postoperative atrial fibrillation; CV, cardiovascular; LV, left ventricular; HR, hazard ratio; OR, odds ratio; MI, myocardial infarction; Prev., prevalence.

Enhanced ECG-based heart rhythm monitoring has further clarified the incidence and implications of AF. A cohort of 317 patients undergoing abdominal aortic aneurysm repair or peripheral bypass underwent 72 hours of continuous ECG monitoring (extended to 30 days), during which up to 4.7% developed new-onset AF [34]. Nevertheless, most episodes were asymptomatic and occurred postoperatively, indicating their transience, as nearly all patients returned to normal heart rhythm within a month [34]. Despite this, the risk of AF was linked to a higher risk of heart problems both around the time of surgery (adjusted HR 6.0, 95% CI 2.4–15.0) and within one year (HR 4.2, 95% CI 2.1–8.8). Notably, many patients also showed signs of heart injury, such as myocardial ischemia or elevated troponin, preceding the onset of AF [34]. This reaffirms that AF can largely be a downstream reaction to myocardial stress and ischemia occurring perioperatively as opposed to an isolated arrhythmic event [34].

Similar patterns have been observed in other vascular cohorts. In a study of 513 vascular surgery patients, about 10% developed new arrhythmia problems, with half of these being characterized as AF. Older age and weaker heart function were the key risk factors, and the development of a perioperative arrhythmia doubled the risk of future cardiovascular problems (HR 2.2) [11]. Moreover, among 186 patients having carotid surgery, new AF occurred in 3.8% of patients, all in the postoperative period. Low BP during surgery (90/60 mmHg) was the strongest predictor (OR 9.6, 95% CI 1.9–47.4) of AF, and patients with both hypotension and AF had a higher risk of stroke [35].

### 3.4 Intraoperative AF in Orthopedic Surgery

Evidence regarding IOAF in orthopedic surgery derives predominantly from hip and knee arthroplasty cohorts (Table 4, Ref. [36,37,38]). Continuous rhythm monitoring has provided substantial insight into the timing of arrhythmic onset. In a study of 583 total hip or knee arthroplasties, patients were continuously monitored with ECG during surgery and for at least 3 hours afterward. New rapid heart rhythms (AF or other supraventricular tachyarrhythmias) occurred in 4.8% of patients [36]. Notably, most of these events (60.7%) began intraoperatively, with 10.7% in the recovery room on the day of surgery [36]. These findings suggest that a significant proportion of AF episodes in orthopedic surgery may arise during the intraoperative phase rather than postoperatively, reinforcing the importance of developing mitigation strategies.

**Table 4.** **Orthopedic surgery studies: design/sample, procedures, monitoring window, incidence, and key risk factors/outcomes**.

Study	Design/n	Procedures	Monitoring window	Incidence	Key risk factors/outcomes
Kahn et al., 1993 [36]	Prospective, n = 1210 (subset n = 583)	THA/TKA	Continuous ECG OR → PACU ≥3 h	AF/SVT 3.1% (4.8% in subset)	Prior AF, age, LA hemiblock, atrial premature beats; high‑risk subgroup incidence 18.2% vs 1.9% in low risk
Khormaee et al., 2018 [37]	Administrative cohort, n = 528,246	Primary THA/TKA	Hospitalization; “new‑onset perioperative AF”	0.7%	New periop AF → 2.7‑fold higher 1‑year ischemic stroke risk; ↑LOS and charges
Leibowitz et al., 2017 [38]	Retrospective, n = 410	Hip fracture repair (age ≥65)	Hospital stay	New AF 3.7%	Periop AF strongest predictor of 1‑year mortality (HR 6.7); the association is not attenuated by antiarrhythmic/anticoagulant use in this small cohort

Abbreviations: n, sample size; THA, total hip arthroplasty; TKA, total knee arthroplasty; ECG, electrocardiogram; OR, operating room; PACU, post-anesthesia care unit; AF, atrial fibrillation; SVT, supraventricular tachycardia; LA hemiblock, left anterior hemiblock (left anterior fascicular block); LOS, length of stay; HR, hazard ratio.

Risk for IOAF in this population was strongly driven by a history of AF, older age, a left anterior hemiblock, and ectopic beats seen on the preoperative ECG. Among patients aged ≥60 years with at least one of these risk factors, the incidence of IOAF was 9.6-fold higher than in patients <60 years without risk factors [36,37]. Moreover, large administrative datasets give a broader view of IOAF; however, they do not pinpoint when exactly it occurs. In New York, among 312,636 total knee arthroplasties (TKAs) and 215,610 total hip arthroplasties (THAs), new AF (not present at admission) happened in 0.7% of patients [36,37]. Those who developed AF were older and had more vascular diseases and higher CHA_2_DS_2_‑VASc scores [37]. IOAF was associated with prolonged hospitalization (TKA 7.8 vs 4.2 days; THA 8.0 vs 4.4 days), increased healthcare costs, and a 2.7-fold higher risk of ischemic stroke within one year.

The prognostic implications of AF are particularly more pronounced in orthopedic populations facing age-related vulnerability. In a study of 410 elderly hip-fracture patients (over 65 years of age) who were in normal rhythm before surgery, 3.7% developed AF during their hospital stay. Additionally, new AF was a strong predictor of poor outcomes: one-year mortality was 60% in patients with AF compared to 19.5% without AF. Furthermore, AF was the strongest independent predictor of death in this group (HR 6.7, 95% CI 2.1–21.4) [38]. It is important to note that surgical variables such as the type of joint, whether one or both joints were replaced, and anesthesia type did not affect the risk within these cohorts.

### 3.5 Intraoperative AF in Neurosurgery

Evidence regarding IOAF in neurosurgical practice remains limited, deriving predominantly from case reports and small patient series. These reports show that AF can emerge both intraoperatively and postoperatively, most often in patients with significant cerebrovascular vulnerability or when pharmacological agents such as adenosine are used.

In the setting of aneurysmal subarachnoid hemorrhage, Malik et al. (2014) [39] described a 46-year-old woman undergoing emergency clipping of an anterior communicating artery aneurysm. She developed IOAF (~200 bpm) when she arrived in the operating room, likely triggered by hypokalemia [39]. She had recurrent AF with severe hypotension 30 minutes into surgery and again on the first postoperative day. Despite attempts to control her rhythm, she later died from a presumed brain infarction due to vasospasm.

Expanding on AF in relation to aneurysm clipping surgery, in a series of 24 clippings using adenosine, Bebawy et al. (2010) [40] observed two patients with brief, stable AF; one resolved on its own, the other with amiodarone, and there were no major complications. Intarakhao et al. (2020) [41] reported 13 episodes of short adenosine-induced asystole in nine patients. One patient with frequent extra atrial beats developed AF after adenosine, which resolved in the ICU with amiodarone [41]. Beyond vascular neurosurgery, brain tumour surgery can additionally trigger AF through neurogenic mechanisms. Joys et al. [42] reported a 64-year-old woman with a sphenoid wing meningioma who developed IOAF (153–160 bpm), even though her preoperative ECG was normal. The arrhythmia persisted during induction but terminated abruptly following neck repositioning, suggesting a mechanistic role for venous congestion, elevated intracranial pressure, or autonomic reflexes [40,41,42].

Deb et al. [43] reported that repeat adenosine dosing during aneurysm clip reconstruction precipitated supraventricular tachycardia (SVT) progressing to AF with persistent hypotension, requiring synchronized cardioversion (100 J) with immediate return to sinus rhythm. Furthermore, Quiroz-Murga et al. [44] described AF with severe low BP after a third adenosine bolus during multiple aneurysm clippings, requiring electrical cardioversion and drugs to support BP; afterward, ECG and troponin were normal, and AF did not recur.

Collectively, these accounts indicate that while IOAF in neurosurgery is rare, it is context-dependent and most commonly occurs in association with vascular interventions (before or during aneurysm clipping either due to the hemorrhage itself or adenosine use) or from extreme patient positioning. Episodes are usually short-lived but can recur. Since no large cohort studies presently exist, the exact incidence and outcomes of intraoperative AF in neurosurgery are unknown.

## 4. Cross-Specialty Risk Factors and Mechanistic Themes

Across surgical populations, IOAF arises from the interplay among patient profile, procedural stress, and acute physiologic perturbations. Large lung‑resection cohorts show IOAF incidences of 1–3%, with risk concentrated in older male patients undergoing more extensive resections, particularly lobectomy with systematic lymph‑node dissection [20,21]. Mechanistically, these studies point toward combined effects of surgical trauma, vagal stimulation, systemic inflammation and autonomic imbalance, with IOAF clustering around periods of intensive mediastinal manipulation and dissection [23,24]. In esophagectomy, arrhythmias, including AF, are even more common, with intraoperative arrhythmia rates around 17% and a high early recurrence rate [23]. The interaction of the following predictors promotes AF: pre‑existing heart disease, increased central venous pressure, impaired pulmonary function, and heavy catecholamine use. Prospective studies demonstrate that both the duration and intensity of mediastinal dissection correlate with hypotension and electrical instability, reinforcing a dose–response relationship between surgical stress and atrial vulnerability [24,25].

Data from vascular surgery portray IOAF as a marker of global cardiovascular vulnerability rather than a locally triggered phenomenon. In vascular surgery cohorts, new‑onset AF during or shortly after major vascular surgery was infrequent (4.7%) but carried a strikingly elevated risk of both early and late cardiovascular events [34]. This was extended by research displaying that IOAF amplifies the adverse impact of intraoperative hypotension during carotid endarterectomy, an intuitively plausible interaction in a population with cerebrovascular disease [35]. Holistically, these observations emphasize that IOAF in vascular surgery is a reaction to depleted physiological reserve rather than an isolated event.

Abdominal and liver‑transplant data, though less comprehensive, delineate IOAF’s role in strongly complicating patients’ postoperative journeys. Even brief IOAF during liver transplantation predicted longer ICU stay and a higher burden of early post‑transplant complications [27]. Case‑based reports across general surgery consistently link IOAF with major shifts in preload/afterload, blood loss, electrolyte changes, or high catecholamine doses [12,32]. Moreover, in orthopedic and neurosurgical practice, IOAF is rare enough that most of what is known arises from IOAF databases (orthopedics) or adenosine‑assisted aneurysm surgery (neurosurgery). In hip and knee arthroplasty, perioperative supraventricular tachyarrhythmias cluster in older patients with prior AF or conduction disease, but timing relative to the operation is poorly resolved [36]. In neurosurgery, AF appears almost exclusively as a rare complication of high‑dose or repeated adenosine boluses, underscoring the role of pharmacologic triggers in a structurally normal heart [40,41,43,44]. Collectively, exercising surgical caution, maintaining patient stability, and accounting for patient comorbidities prior to surgery are fundamental to mitigating intraoperative and postoperative complications associated with IOAF development.

Overall, pathophysiologically, IOAF likely reflects the interaction of a vulnerable atrial substrate with acute perioperative triggers. Autonomic imbalance can promote AF by dysregulating both the sympathetic and parasympathetic systems, altering atrial electrophysiology and increasing arrhythmia susceptibility [45]. Sympathetic activation is highly relevant intraoperatively, as catecholamine surges associated with surgical stress, pain, and hemodynamic instability have been implicated in both AF initiation and maintenance [46]. Atrial stretch from abrupt preload or afterload shifts may further facilitate AF by promoting structural remodeling, including atrial enlargement and fibrosis [47]. Inflammation also strengthens the AF substrate by accelerating electrical and structural atrial remodeling through pro-inflammatory cytokines and related signaling pathways [48].

**Fig. 2.**
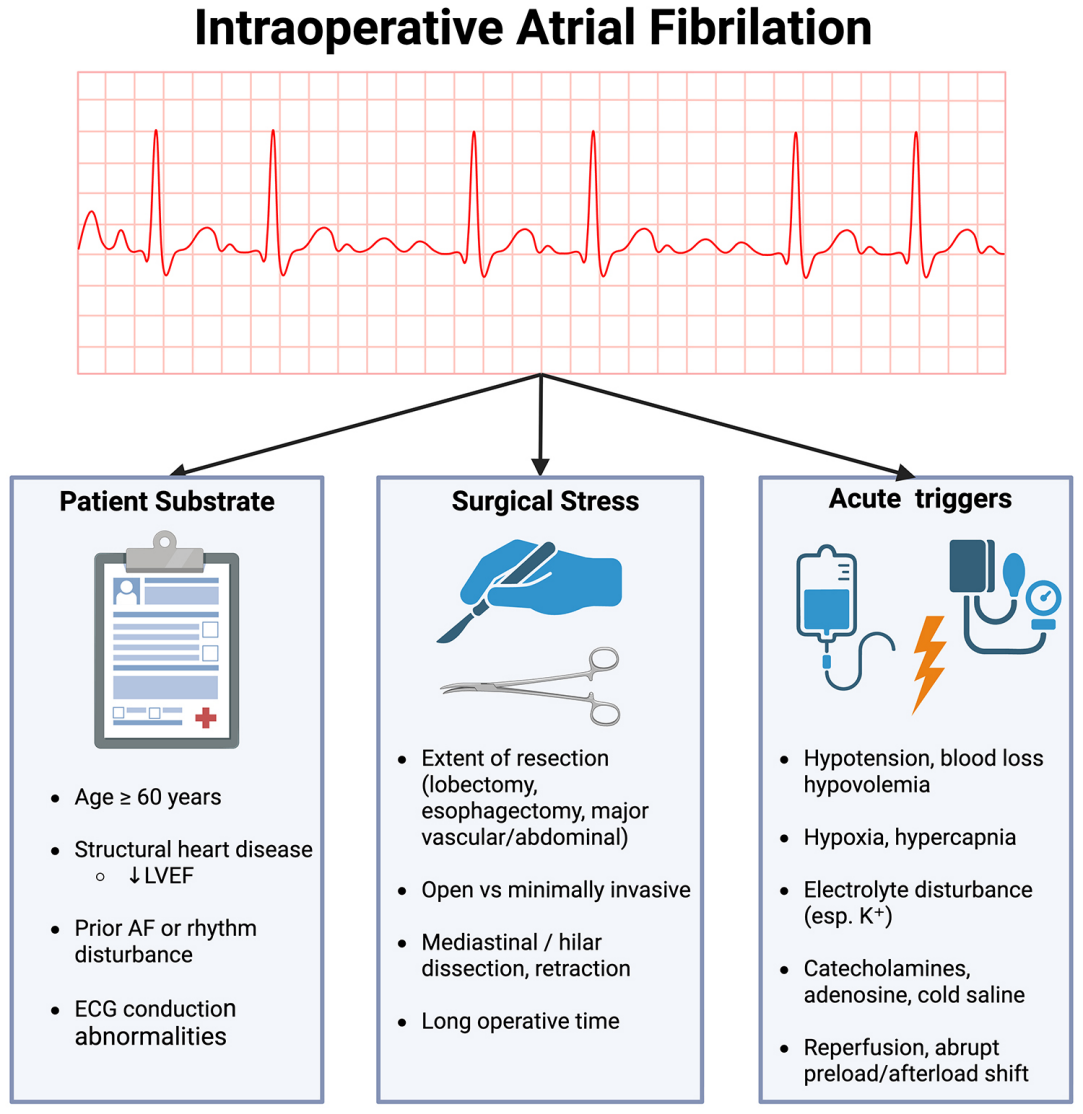
**Three domains that predispose to intraoperative atrial fibrillation across non-cardiac surgery types**. Across multiple non-cardiac surgery types, three themes consistently emerge that increase the likelihood of atrial fibrillation during the intraoperative period, as illustrated: (1) Patient substrate (age ≥60 years, structural heart disease/↓left ventricular ejection fraction (LVEF), prior atrial fibrillation (AF) or rhythm disturbance, baseline electrocardiogram (ECG) conduction abnormalities), (2) Surgical stress (greater extent of resection, including lobectomy, esophagectomy, major vascular or abdominal surgery and open vs minimally invasive approach) and (3) Acute intraoperative triggers such as hypotension/blood loss/hypovolemia, hypoxia/hypercapnia, electrolyte disturbances, and reperfusion or abrupt preload/afterload shifts.

As illustrated by Fig. 2, across all specialties, three broad and recurring risk domains emerge: (1) Patient substrate–age, prior AF, structural heart disease, and conduction abnormalities on ECG [20,36]. (2) Procedure, surgical stress, such as the extent of resection, open vs minimally invasive approach, mediastinal or hilar manipulation, operation time [20,21,23]. (3) Acute physiologic triggers include hypotension, hypovolemia, anemia, electrolyte imbalance, catecholamines, and, in neurosurgery, adenosine [24,27,35,44].

IOAF presents differently in cardiac and non-cardiac surgeries. AF affects 32.3% of patients after cardiac surgery, and it usually occurs a couple of days after surgery due to direct atrial injury, reduced blood flow, cardiopulmonary bypass, severe inflammation, or irritation of heart tissue [49]. In contrast, IOAF in non-cardiac surgery is less common (generally <5%) and occurs during or immediately after surgery, usually in response to transient, reversible stressors [50]. Nevertheless, both contexts share common risk factors, such as advanced age, prior AF, and structural heart disease, supporting the concept that non-cardiac IOAF often unmasks an intrinsically vulnerable atrium. In comparison, in non-cardiac surgeries, IOAF is less common (1–5%), but if it does occur, it typically happens during or right after the surgery [51]. Unlike cardiac surgery, where AF is caused by lasting heart damage, non-cardiac surgery AF is usually caused by short-term reversible problems like changes in BP, low oxygen, high levels of the stress hormones, or if the tissues nearby are irritated [52].

## 5. Definition and Monitoring Challenges

A major barrier to synthesis is definitional heterogeneity. In the literature, the terms “preoperative AF”, “perioperative AF”, and “intraoperative AF” are often used interchangeably. Further, even among IOAF‑focused studies, definitions vary substantially. For instance, Tong et al. (2021) [21] defined IOAF as AF ≥1 minute on ECG from induction to the end of thoracoscopic lung surgery. Wu et al. (2012) [20] also restricted AF to the intraoperative period but did not require a minimum duration beyond clinical recognition. Winkel et al. (2009) [34] labelled AF detected within 72 hours by continuous monitoring as “during vascular surgery”, even though most episodes arose postoperatively in the ICU or ward. Esophagectomy and general‑surgery studies often pool intraoperative and early postoperative arrhythmias together [23,24]. Moreover, some studies apply general AF definitions (e.g., ≥30 seconds of irregularly irregular rhythm without P waves), whereas others include any supraventricular arrhythmia under a single “arrhythmia” endpoint. This variability complicates cross‑study comparisons and limits predictive modelling. Adoption of standardized outcome definitions, such as the European Perioperative Clinical Outcome (EPCO) criteria, would enhance comparability and improve risk-stratification research [21,53].

## 6. Clinical Implications and Management Considerations

Despite the heterogeneity, certain management patterns are consistent and can be synthesized into pragmatic intraoperative principles. Firstly, continuous ECG is essential in moderate‑ to high‑risk non-cardiac surgery, as IOAF episodes are only detectable with such monitoring [15,54]. Studies that used continuous intraoperative and early postoperative ECG reported higher AF detection than those relying solely on symptomatic episodes [21,34]. Immediate correction of reversible triggers, like hypotension, hypoxia, bleeding, electrolyte disturbances, and drug effects, should precede decisions regarding rate or rhythm control [23,24,27,44]. This aligns with acute AF guidance for hospitalized patients [17,19].

When BP is acceptable, beta‑blockers or calcium‑channel blockers are typically used for rate control, with many episodes terminating spontaneously [20,21,32]. Synchronized electrical cardioversion is favoured in hemodynamically unstable IOAF or when AF threatens myocardial perfusion or surgical exposure [12,44].

Even when brief and self‑terminating, IOAF consistently predicts longer ICU and hospital stay and greater risk of early complications [21,27,34]. Given ample data that IOAF is associated with increased long‑term stroke and mortality, it is reasonably consistent with AHA and ACC guidelines to arrange cardiology follow‑up, reassess CHA_2_DS_2_‑VASc, and consider long‑term anticoagulation once surgical bleeding risk is acceptable [4,19,38,55,56]. The management strategies of IOAF differ between cardiac and non-cardiac surgeries. In cardiac surgeries, AF is common, so it is often prevented preoperatively using beta-blockers, amiodarone, or pacing strategies [52,57,58]. In comparison, in non-cardiac surgery, routine prophylaxis is not recommended; instead, emphasis is placed on vigilant monitoring and physiologic optimization [51].

## 7. Limitations

There are several limitations to consider when interpreting the findings of this review. Firstly, the evidence on IOAF for non-cardiac surgeries is largely observational and uneven; much of the significant data comes from thoracic and vascular cohorts. Small studies and case reports also contribute to the findings, limiting causal inference and generalizability. Moreover, the lack of standardization in the definition and detection of IOAF complicates analysis, as study categorizations vary across time windows (IOAF vs broader “perioperative AF”), episode intensity, and arrhythmia definitions. The presence of other confounding factors also plays a role, as many studies do not adequately adjust for factors such as cardiac history (prior AF/structural disease), anesthetic approach, medication use, electrolyte and ionic disturbances, and whether AF is primary or a result of other surgical complications. Lastly, outcome reporting and follow-up protocols are inconsistent, with variable descriptions of management strategies (rate/rhythm control and timing of anticoagulation) that create uncertainty when attempting to establish conclusions on optimal long-term treatment and risk mitigation. Future work would benefit from standardized IOAF definitions and monitoring windows, prospective multicenter designs with granular intraoperative data, and longer follow-up to inform procedure-specific pathways. Without standardized definitions, the true incidence, timing, and clinical significance of IOAF remain difficult to quantify, limiting both research synthesis and clinical translation.

## 8. Conclusion

From an electrophysiological perspective, atrial fibrillation occurring during non-cardiac surgery remains an under-defined and inconsistently monitored complication with notable clinical significance. Available evidence supports a structured intraoperative approach: rapid assessment of hemodynamic stability, confirmation of AF, systematic correction of reversible triggers, and individualized rate or rhythm control. Hemodynamic instability mandates urgent cardioversion. Decisions regarding anticoagulation require careful balancing of thromboembolic and procedure-specific bleeding risks. This review presents recommendations to inform the establishment of an intraoperative approach for managing IOAF, based on available perioperative evidence. The management framework is multifaceted; it begins with rapid assessment of hemodynamic stability, followed by the confirmation of AF, and systematic alteration of reversible triggers (bleeding, hypoxia, acid–base and electrolyte disturbances, catecholamine exposure, and surgical manipulation). Rate or rhythm control should be individualized to ventricular function and procedural context, while instability should be managed by urgent cardioversion. In terms of anticoagulation, a holistic balance between mitigating stroke risk and procedure-specific bleeding risk must be achieved. Future research should focus on standardized IOAF definitions, risk-based monitoring, and further elucidating procedure-specific pathways.
